# Loading characteristics data applied on osseointegrated implant by transfemoral bone-anchored prostheses fitted with state-of-the-art components during daily activities

**DOI:** 10.1016/j.dib.2022.107936

**Published:** 2022-02-10

**Authors:** Laurent Frossard, Stefan Laux, Marta Geada, Peter Paul Heym, Knut Lechler

**Affiliations:** a YourResearchProject Pty Ltd, Brisbane, QLD, Australia; b Griffith University, Gold Coast, QLD, Australia; c APC Prosthetics Pty Ltd, Alexandria, NSW, Australia; d Sum Of Squares - Statistical Consulting, Leipzig, Germany; e ÖSSUR, Reykjavik, Iceland

**Keywords:** Artificial limb, Bone-anchored prosthesis, Direct skeletal attachment, Osseointegrated implants, Osseointegration, Prosthetic knees, Prosthetic feet loading, Kinetics

## Abstract

The data in this paper are related to the research article entitled “Load applied on osseointegrated implant by transfemoral bone-anchored prostheses fitted with state-of-the-art prosthetic components” (Frossard et al. Clinical Biomechanics, 89 (2021) 105457. DOI: 10.1016/j.clinbiomech.2021.105457). This article contains the overall and individual loading characteristics applied on transfemoral press-fit osseointegrated implant generated by bone-anchored prostheses fitted with state-of-the-art components during daily activities (i.e., microprocessor-controlled Rheo Knee XC knee, energy-storing-and-returning Pro-Flex XC or LP feet (ÖSSUR, Iceland)). Confounders of the loads are presented. The load profiles are characterized by the loading patterns, loading boundaries and loading local extrema of the forces and moments applied during straight-level walking, ascending and descending ramp and stairs at self-selected comfortable pace. The confounders of the loading information as well as new insights into inter-participants variability of loading patterns, loading boundaries and loading local extrema can inform the design of subsequent cross-sectional and longitudinal studies as well as literature reviews and meta-analyzes. The loading datasets are critical to clinicians and engineers designing finite element models of osseointegrated implants (e.g., medullar and percutaneous parts) and prosthetic components, algorithms capable to recognize the loading patterns applied on a residuum during daily activities, as well as clinical trials assessing the effects of particular prosthetic care interventions. Altogether, these datasets provide promoters of prosthetic care innovations with valuable insights informing the prescription of advanced prosthetic components to the growing population of individuals suffering from lower limb loss choosing bionics solutions. Online repository contains the files: https://data.mendeley.com/datasets/gmsyv97cpc/1

## Specifications Table

**Table:** 

Subject	Biomedical Engineering
Specific subject area	Design of prosthesis for individuals fitted with osseointegrated implant
Type of data	Table, graph
How data were acquired	Thirteen participants ambulated with an instrumented bone-anchored prosthesis made of tube and/or offset connector, transducer, Rheo Knee XC, Pro-Flex XC or LP feet (ÖSSUR, Iceland) and their own footwear. The tri-axial transducer measured directly and sent the loading data wirelessly to laptop nearby.
Data format	Raw, analyzed
Parameters for data collection	The forces and moments applied on and around the mediolateral, anteroposterior and long axes of transfemoral osseointegrated implant were recorded with sampling frequency set at 200 Hz and an accuracy better than 1 N and 1 Nm, respectively.
Description of data collection	Participants with transfemoral amputation conducted up to five trials of standardized daily activities (e.g., level walking, ascending and descending stairs and ramp) at self-selected speed using a instrumented bone-anchored prostheses.
Data source location	APC Pty Ltd, Alexandria, NSW, Australia
Data accessibility	Data is with this article. Transparency data including all tables presented in this article can be found in online from: Repository name: Mendeley Data Data identification number: 10.17632/gmsyv97cpc.1 Direct URL to data: https://data.mendeley.com/datasets/gmsyv97cpc/1
Related research article	L. Frossard, S. Laux, M. Geada, P.P. Heym, K. Lechler, Load applied on osseointegrated implant by transfemoral bone-anchored prostheses fitted with state-of-the-art prosthetic components, Clin Biomech (Bristol, Avon) 89 (2021) 105,457, DOI: 10.1016/j.clinbiomech.2021.105457[1].

## Value of the Data

• The loading profile applied on transfemoral osseointegrated implants by bone-anchored prostheses fitted with state-of-the-art prosthetic components presented here were characterized by several datasets including the confounders as well as the loading patterns, loading boundaries and loading local extrema of the forces and moments applied during straight-level walking, ascending and descending ramp and stairs [2], [3], [4], [5].
• These datasets are essential for promoters of prosthetic care innovations (e.g., users, clinicians, engineers, scientists, administrators) because they provide valuable insights supporting the prescription of advanced prosthetic components to the growing population of individuals suffering from lower limb loss choosing bionics solutions [6], [7], [8], [9], [10], [11].
• The confounders of the loading information as well as the new evidence of inter-participants variability of loading patterns, loading boundaries and loading local extrema are required to inform providers of prosthetic care who will design subsequent cross-sectional and longitudinal studies (e.g., statistical planning, power calculation) as well as subsequent literature reviews and meta-analyzes [12], [13], [14].
• More precisely, the loading datasets are critical to clinicians (e.g., rehabilitation specialists) and engineers (e.g., manufacturers of components) designing finite element models of prosthetic components and osseointegrated implants parts (e.g., medullar and percutaneous parts), algorithms capable to recognize the loading patterns applied on a residual limb during daily activities, as well as clinical trials testing effects of particular interventions (e.g., design-based selection of components, alignment of prostheses) [15], [16], [17].

## 1 Data Description

**Fig. 1:**
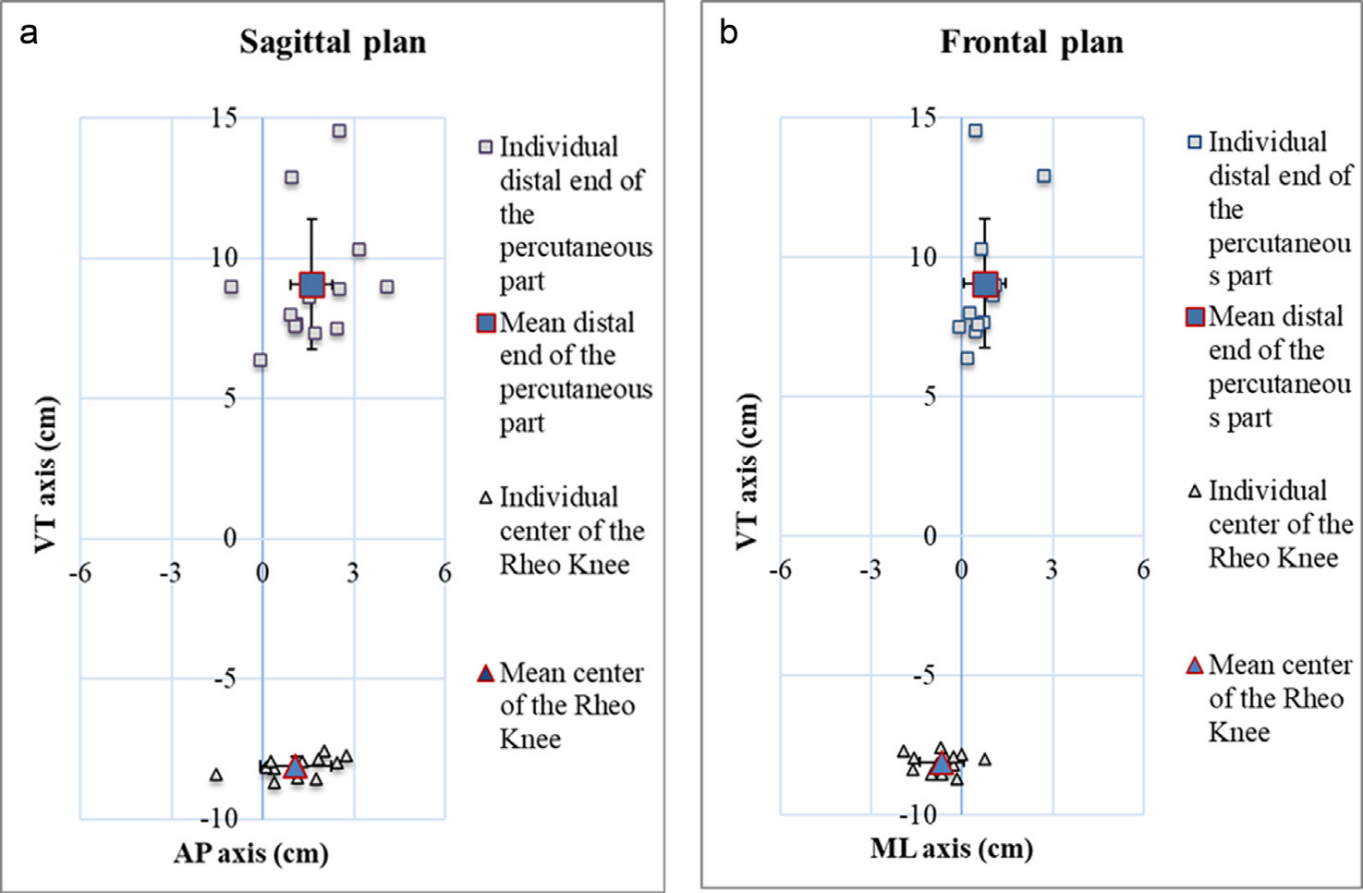
Position of the distal end of the percutaneous part and the center of the Rheo Knee in relation to the origin of coordinate system of iPecsLab's transducer (RTC Electronics, USA) on the antero-posterior (AP), medio-lateral (ML) and vertical (VT) axes of the sagittal and frontal planes.

The confounders of the loading characteristics data including the selection criteria as well as the demographics, amputation, and residuum information as well as prosthesis and alignment of transducer are presented in Table 1, Table 2, Table 3, Table 4, Table 5, Table 6, Table 7 and Fig. 1, respectively. The confounders of the study design including non-experimental setup and number of gait cycles analyzed information are presented in Tables 8 and 9, respectively.

The loading boundaries corresponding to the overall minimum and maximum of forces and moments applied on the implant expressed in units and percentage of the bodyweight were presented in Table 10.

The mean and standard deviation of the pattern as well as the dispersion and mean for up to three local extrema of forces and moments during walking, ascending and descending ramp and stairs are presented in Figs. 2, 4, 6, 8, and 10, respectively.

The box plots of magnitude of up to three local extrema of forces and moments during walking, ascending and descending ramp and stairs are presented in Figs. 3, 5, 7, 9 and 11, respectively.

The Mendeley Data include a spreadsheet and a report providing the confounders (e.g., selection criteria, demographics, individual amputation and residuum information, individual prosthesis and alignment of transducer data, description of non-experimental setup, number of gait cycles) and overall loading boundaries (e.g., minimum and maximum of forces and moments) of the loading data during level walking, ascending and descending ramp and stairs.

### Confounders

**Table 1:** Selection criteria including inclusion and exclusion criteria applied for the recruitment and selection of participants using unilateral transfemoral bone-anchored prosthesis fitted with state-of-the-art components.

**Inclusion criteria**
1. To be fitted with osseointegrated fixation more than 6 months prior testing. 2. To be fully rehabilitated. 3. To have a clearance of at least 6 cm between percutaneous part of the fixation (e.g., abutment, dual cone) and prosthetic knee joint to fit the transducer. 4. To be able to be fitted with one of the nominated ÖSSUR components. 5. To be willing to participate to this project of research. 6. To be willing to comply with protocol. 7. To be able to walk 200 m independently with prosthesis. 8. To be between 18–80 years of age. 9. To be free of infection on the day of the recording session.
**Exclusion criteria**
1. To have bilateral amputation. 2. To have self-reported pain level greater than 4 out of 10 at study outset. 3. To have experienced a fall within the last 8 weeks before assessment. 4. To have mental illness or intellectual impairment. 5. To not be able to give informed consent. 6. To have injuries involving contralateral (intact) limb. 7. To present signs of infection 2 weeks prior testing session. 8. To have major uncorrected visual deficit. 9. To have history of epilepsy or recurrent dizziness.

**Table 2:** Overall and individual demographics information for cohorts of 13 participants fitted with state-of-the-art Rheo XC knee and Pro-Flex XC or LP feet. M: Male, F: Female, BMI: Body Mass Index. (1) Body mass without prosthesis, (2) Calculated based on body mass without prosthesis.

	Demographics				
	Gender	Age	Height	Mass (1)	BMI (2)
Participant	(M/F)	(Yrs)	(m)	(kg)	(kg/m2)
1	M	30	1.88	103.0	28.0
2	M	34	1.80	102.0	30.2
3	M	55	1.86	110.5	30.8
4	M	47	1.76	74.0	22.8
5	M	59	1.81	84.0	24.6
6	M	61	1.75	91.0	28.6
7	M	54	1.65	80.0	28.1
8	M	66	1.70	82.5	27.3
9	M	58	1.94	83.5	21.3
10	F	63	1.71	54.0	17.3
11	M	59	1.78	69.0	20.7
12	M	81	1.83	117.5	34.0
13	F	70	1.70	71.0	23.4

**Mean**		**57**	**1.78**	**86.3**	**25.9**
**SD**		**14**	**0.08**	**18.0**	**4.7**

**Table 3:** Overall and individual amputations and residuum information for cohorts of 13 participants fitted with state-of-the-art Rheo XC knee and Pro-Flex XC or LP feet. TR: Trauma, TU: Tumor, IN: Infection, OT: Other, L: Left, R: Right, AMP: amputation, BAP: Bone-anchored prosthesis, %SND: Percentage of sound limb.

Participant	Amputation				Residuum	
	Cause	Side	Time since AMP	Time since BAP	Length	Length
		(L/R)	(Yrs)	(Yrs)	(cm)	(%SND)
1	TR	L	8.7	6.01	32	64
2	TR	R	9.1	0.68	15	38
3	TU	L	15.6	3.51	27	57
4	TR	R	30.8	4.69	23	51
5	IN	L	0.4	0.16	28	65
6	TR	R	14.9	2.85	27	59
7	TR	R	11.5	1.93	30	75
8	TR	R	65.7	0.56	25	60
9	TR	L	5.6	1.33	35	66
10	TR	R	41.9	3.00	29	64
11	TU	R	0.7	0.51	28	62
12	IN	R	13.8	1.09	32	71
13	TR	L	1.5	0.76	38	84

**Mean**			**16.94**	**2.08**	**28**	**63**
**SD**			**18.87**	**1.81**	**6**	**11**

**Table 4:** Individual connection between the percutaneous part of the osseointegrated implant including or not a tube and/or an offset adapter (i.e., no tube and no adapter: 15%, a tube and an adapter: 8%, a tube and no adapter: 8%, no tube and an adapter: 69%) and the usual knee (i.e., N/A: 31%, Rheo Knee, OSSUR: 8%, Genium, Ottobock: 31%, 3R80, Ottobock: 15%, C-Leg, Ottobock: 15%) and feet (i.e., N/A: 38%, Vary Flex, OSSUR: 8%, Triton Heavy Duty, Ottobock: 8%, Triton, Ottobock: 15%, Pro-Flex LP, OSSUR: 8%, Trias, Ottobock: 23%) or the instrumented prosthesis fitted with state-of-the-art knee (i.e., Rheo Knee XC, OSSUR: 100%) and feet (i.e., Pro-Flex XC, OSSUR: 69%, Pro-Flex LP, OSSUR: 31%) for the cohort of 13 participants. N/A: Not available.

Participant	Connector		Usual		Instrumented	
	Tube	Offset Adapter	Knee	Ankle	Ankle	Footwear
1	Yes	No	N/A	N/A	XC	Running shoes
2	No	Yes	N/A	N/A	XC	Running shoes
3	Yes	Yes	N/A	N/A	XC	Running shoes
4	No	No	Rheo Knee	Vary Flex	LP	Sandals
5	No	No	Genium	Triton Heavy Duty	XC	Running shoes
6	No	Yes	N/A	N/A	XC	Running shoes
7	No	Yes	Genium	Triton	LP	Mountain boots
8	No	Yes	3R80	N/A	XC	Running shoes
9	No	Yes	Genium	Triton	XC	Flat shoes
10	No	Yes	Genium	Pro-Flex LP	LP	Flat shoes
11	No	Yes	3R80	Trias	XC	Running shoes
12	No	Yes	C-Leg	Trias	XC	Running shoes
13	No	Yes	C-Leg	Trias	LP	Soft Shoes

**Table 5:** Individual alignment of the instrumented bone-anchored prosthesis fitted with iPecsLab's transducer (RTC Electronics, USA) state-of-the-art Rheo XC knee and Pro-Flex XC or LP feet and footwear for the cohort of 13 participants. N/A: Not available.

**Table 6:** Position of the distal end of the percutaneous part and the center of the Rheo Knee in relation to the origin of coordinate system of iPecsLab's transducer (RTC Electronics, USA) on the antero-posterior (AP), medio-lateral (ML) and vertical (VT) axes.

Participant	distal end of the percutaneous part			center of the rheo knee		
	AP	ML	VT	AP	ML	VT
	(cm)	(cm)	(cm)	(cm)	(cm)	(cm)
1	1.53	1.00	8.63	1.12	–0.54	–7.89
2	3.17	0.65	10.31	1.13	–0.68	–8.52
3	–0.08	0.17	6.37	2.74	–1.94	–7.72
4	1.08	0.71	7.66	2.04	–0.70	–7.56
5	–1.04	1.03	9.01	–1.55	–1.61	–8.39
6	1.73	0.44	7.34	0.38	–0.28	–8.19
7	1.06	0.52	7.61	1.82	–0.02	–7.83
8	2.52	0.83	8.93	0.38	–0.17	–8.68
9	2.42	–0.08	7.51	0.13	–0.81	–8.14
10	0.95	2.69	12.92	1.77	–1.01	–8.54
11	2.53	0.43	14.57	1.30	–1.58	–7.95
12	4.09	1.08	9.01	0.27	–0.30	–7.91
13	0.90	0.26	8.01	2.45	0.73	–7.99

**Mean**	**1.61**	**0.75**	**9.07**	**1.08**	**−0.69**	**−8.10**
**SD**	**1.36**	**0.68**	**2.32**	**1.16**	**0.73**	**0.35**

**Table 7:** Individual position of the distal end of the percutaneous part and the center of the Rheo Knee in relation to the origin of iPecsLab's transducer (RTC Electronics, USA) on the antero-posterior (AP), medio-lateral (ML) and vertical (VT) axes of the front and side views in the image (ICS) and transducer (TCS) coordinate systems.

### Overall loading data

**Table 8:** Description of public facilities used for ecological direct measurement of loading applied on osseointegrated fixation by bone-anchored prostheses fitted state-of-the-art components during daily activities.

Activities	State-of-the-art components
**Straight level walking**	
Location	Outdoor
Length (m)	13.00
**Ascending and descending ramp**	
Location	Outdoor
Length (m)	4.11
Incline (deg)	3.77
Height of handrail (cm)	72.00
**Ascending and descending stairs**	
Location	Outdoor
Number of steps	9
Height of step (cm)	17.00
Depth of step (cm)	26.00
Width of step (cm)	114.00
Height of handrail (cm)	70.00

**Table 9:** Breakdown of number of gait cycles analyzed for the cohorts of participants fitted advanced state-of-the-art components during five activities of daily living.

Activity	Number of gait cycles analyzed
Straight-level walking	347
Ascending ramp	252
Descending ramp	268
Ascending stairs	236
Descending stairs	180
**Total**	**1283**

**Table 10:** Loading boundaries including the overall minimum and maximum of forces and moments applied on and around the osseointegrated implant expressed in units and percentage of the bodyweight (%BW).

	Minimum		Maximum	
Forces	(N)	(%BW)	(N)	(%BW)
Long axis	–298	–28	161	1322
Antero-posterior axis	–358	–31	34	388
Medio-lateral axis	–56	–7	16	133
Moments	(Nm)	(%BWm)	(Nm)	(%BWm)
Long axis	–22	–2	2	20
Antero-posterior axis	–52	–6	3	24
Medio-lateral axis	–67	–9	11	88

### Level walking

#### Detection of local extrema

**Fig. 2:**
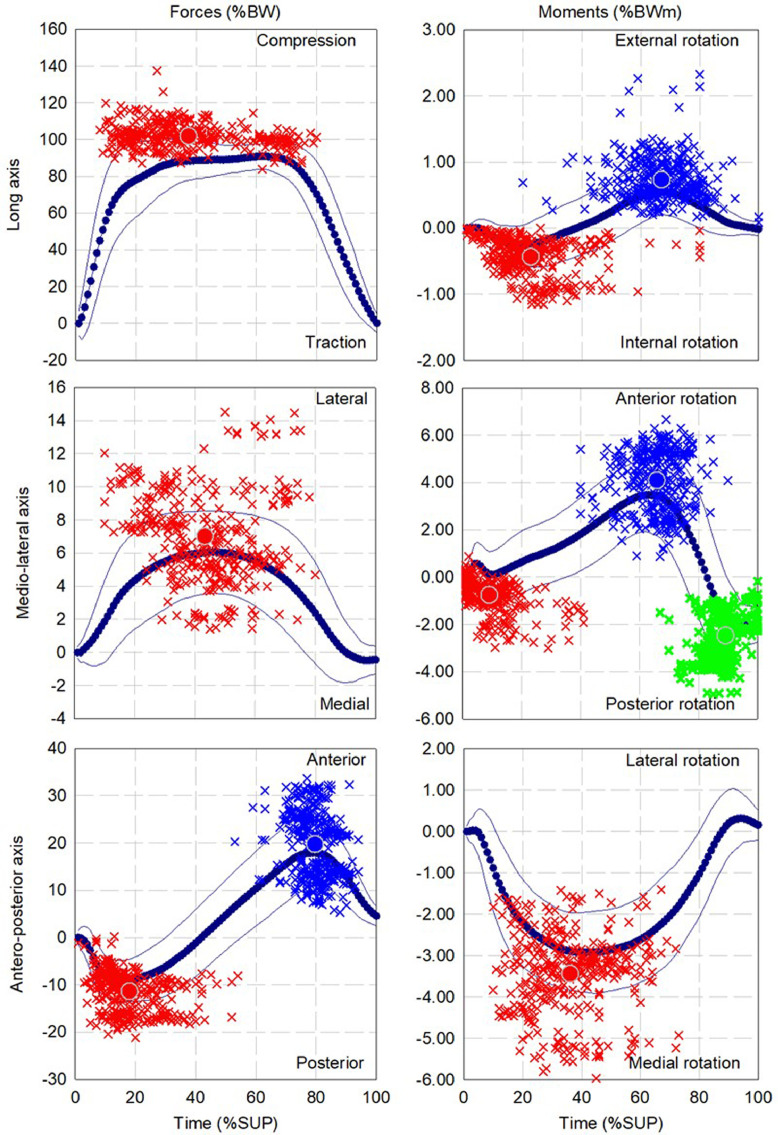
Dispersion (cross) and average (circle) for first (red), second (Blue) and third (green) local extrema of forces and moments for cohort of participants fitted with components (13 participants, 347 gait cycles) during walking.

#### Characteristics of local extrema

**Fig. 3:**
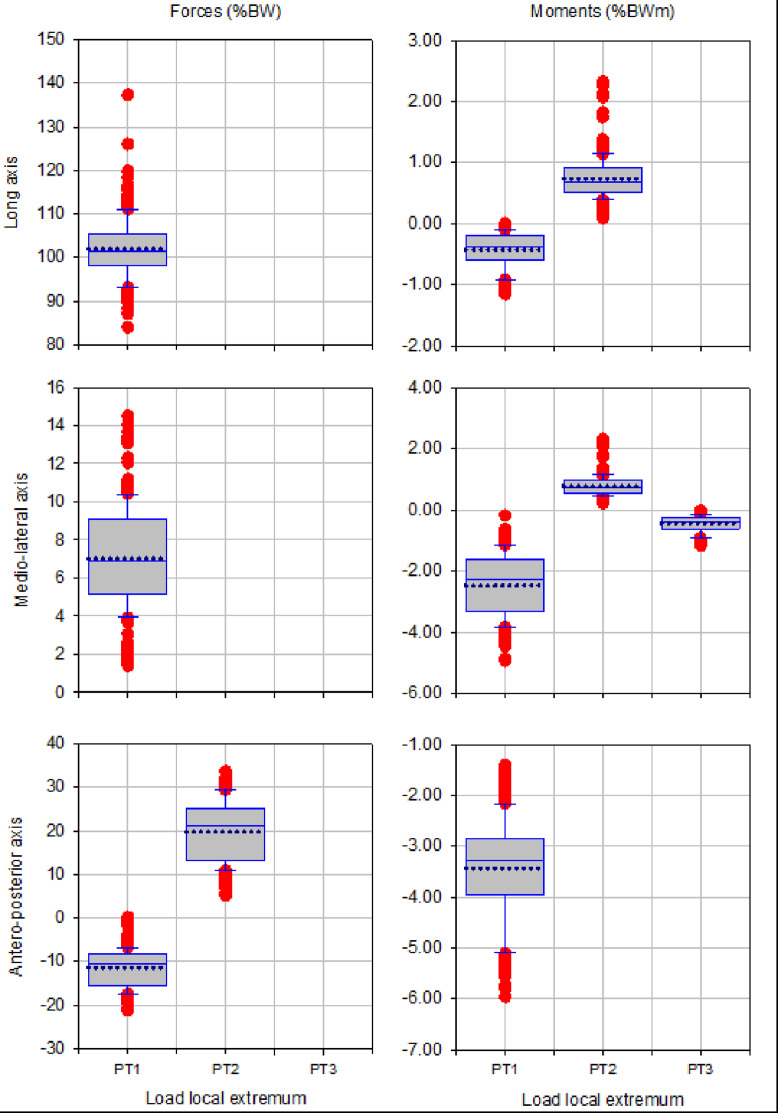
Box plots showing low and high 95% confidence interval, mean and outliers of the magnitude of up to three local extrema (PT1, PT2, PT3) of forces and moments applied with state-of-the-art components during walking.

### Ascending ramp

#### Detection of local extrema

**Fig. 4:**
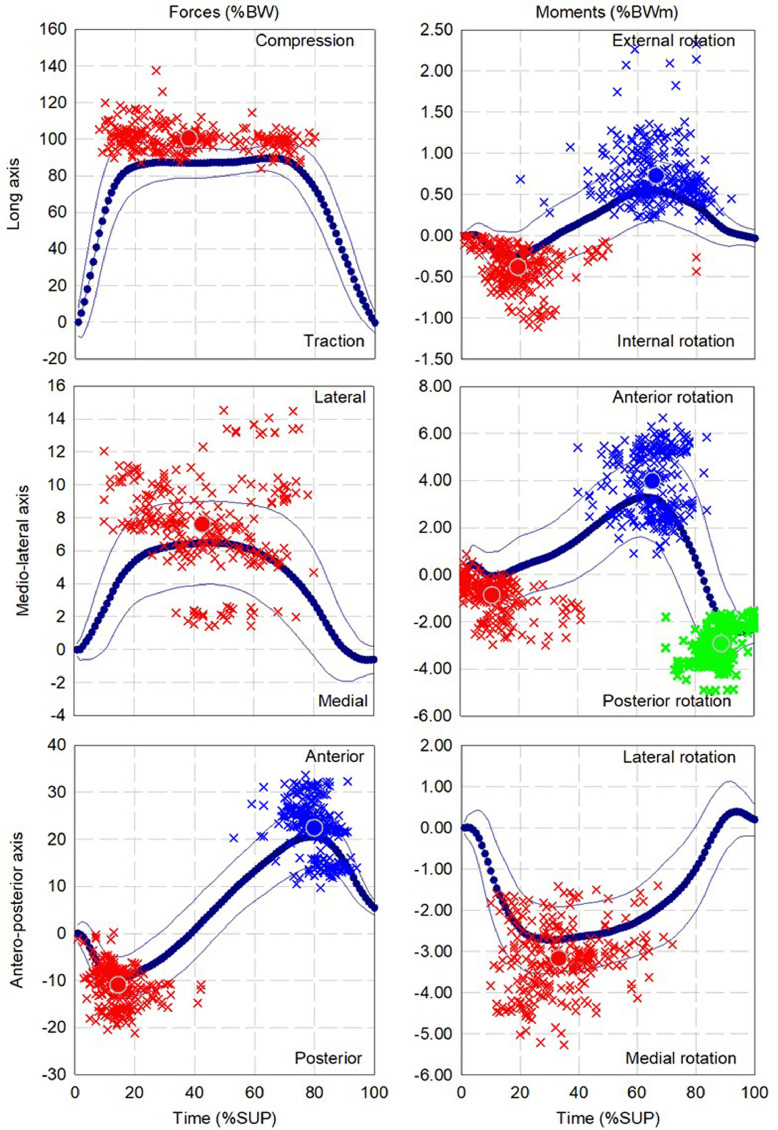
Dispersion (cross) and average (circle) for first (red), second (Blue) and third (green) points of interest of forces and moments for cohort of participants fitted with components (13 participants, 252 gait cycles) during ascending ramp.

#### Characteristics of local extrema

**Fig. 5:**
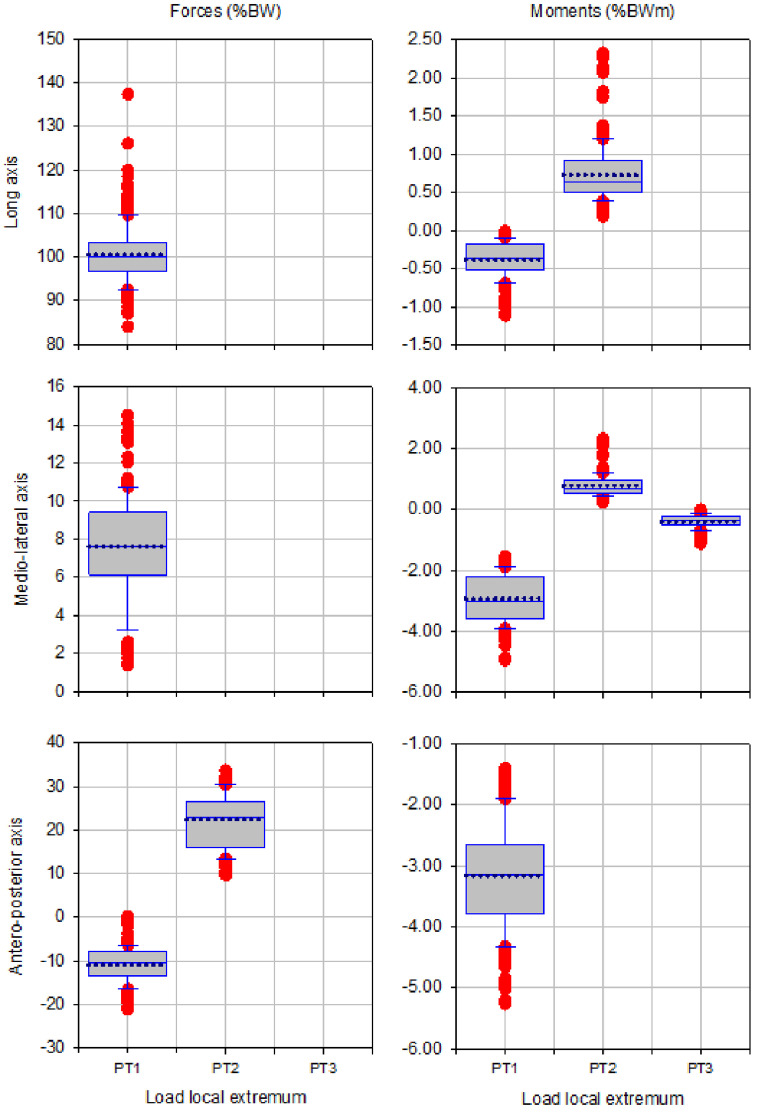
Box plots showing low and high 95% confidence interval, mean and outliers of the magnitude of up to three local extrema (PT1, PT2, PT3) of forces and moments applied with state-of-the-art components during ascending ramp.

### Descending ramp

#### Detection of local extrema

**Fig. 6:**
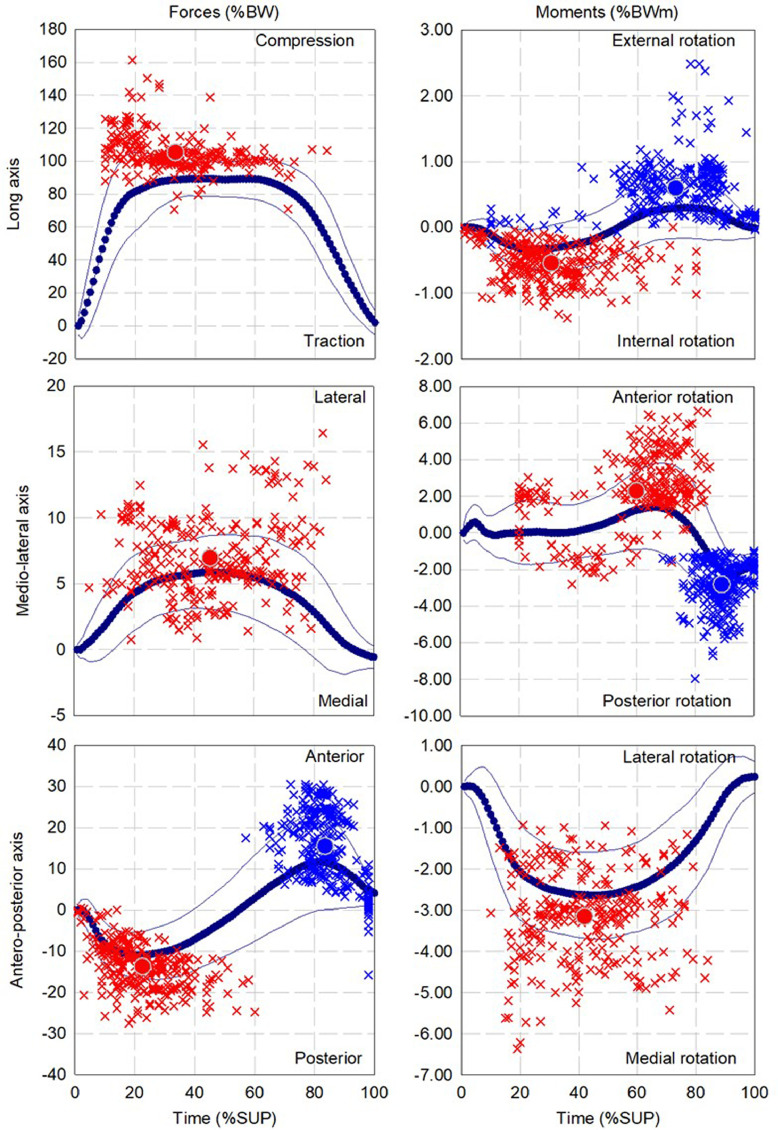
Dispersion (cross) and average (circle) for first (red), second (Blue) and third (green) local extrema of forces and moments for cohort of participants fitted with components (13 participants, 268 gait cycles) during descending ramp.

#### Characteristics of local extrema

**Fig. 7:**
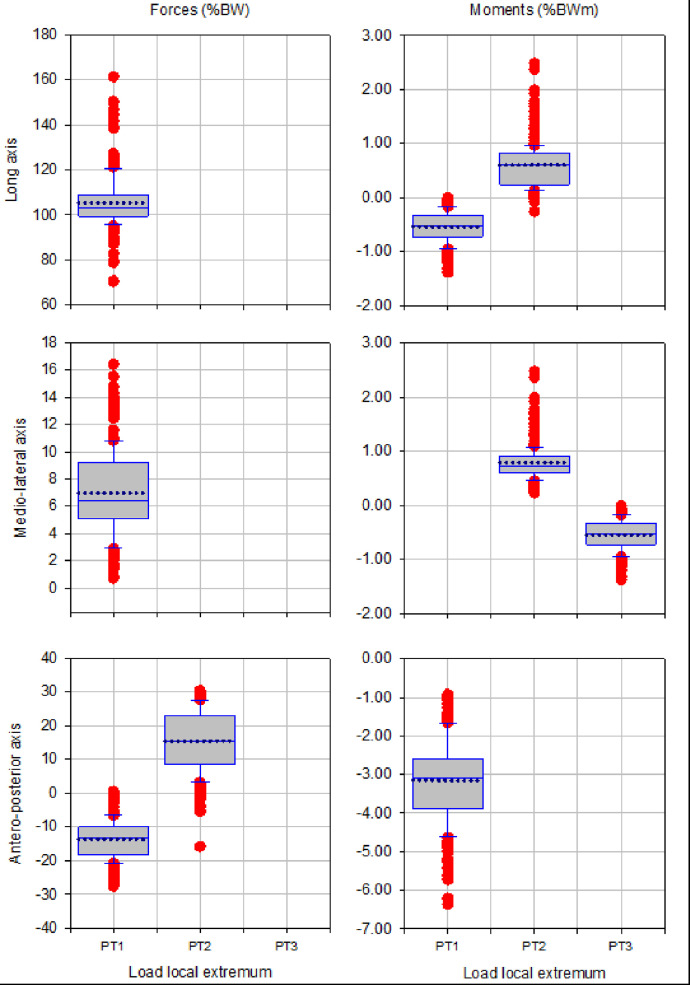
Box plots showing low and high 95% confidence interval, mean and outliers of the magnitude of up to three local extrema (PT1, PT2, PT3) of forces and moments applied with state-of-the-art components during descending ramp.

### Ascending stairs

#### Detection of local extrema

**Fig. 8:**
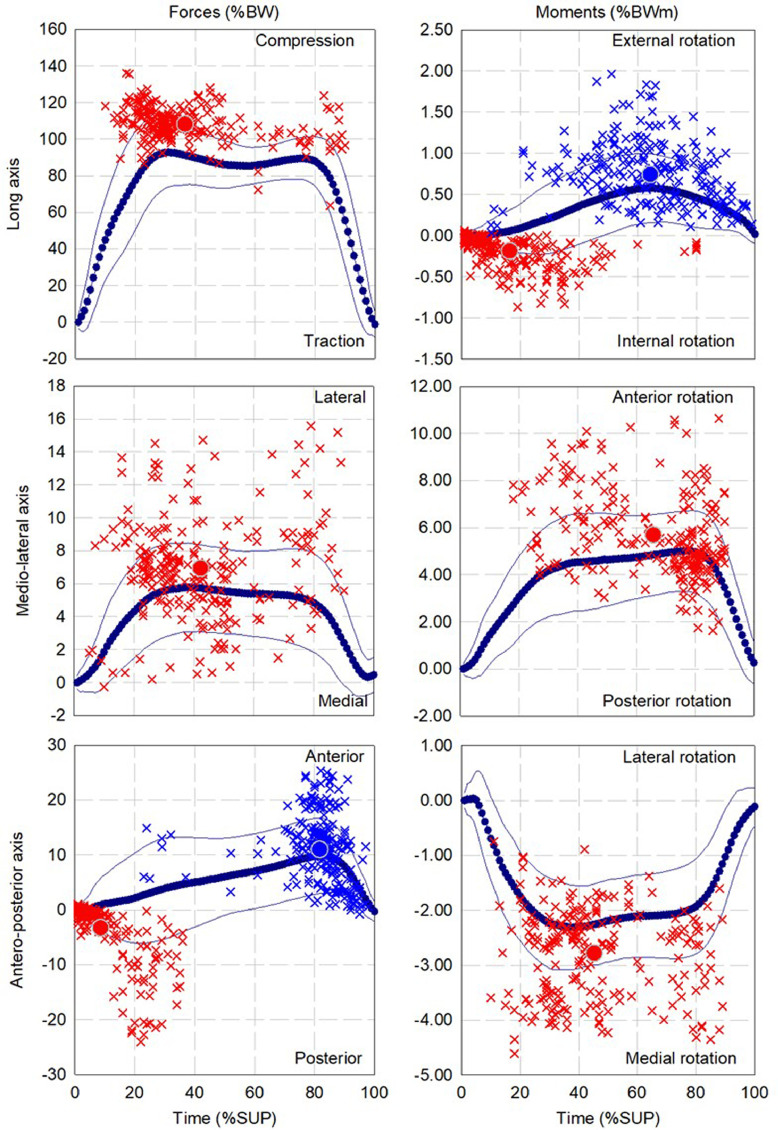
Dispersion (cross) and average (circle) for first (red), second (Blue) and third (green) local extrema of forces and moments for cohort of participants fitted with components (12 participants, 236 gait cycles) during ascending stairs.

#### Characteristics of local extrema

**Fig. 9:**
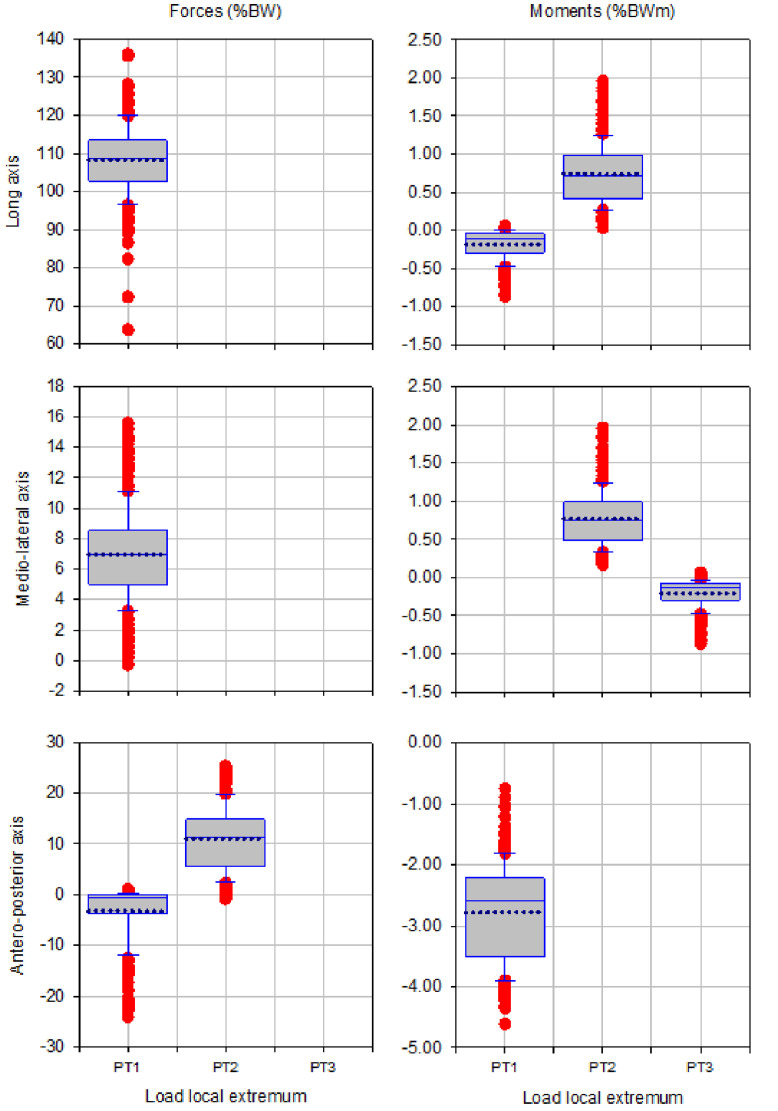
Box plots showing low and high 95% confidence interval, mean and outliers of the magnitude of up to three local extrema (PT1, PT2, PT3) of forces and moments applied with state-of-the-art components during ascending stairs.

### Descending stairs

#### Detection of local extrema

**Fig. 10:**
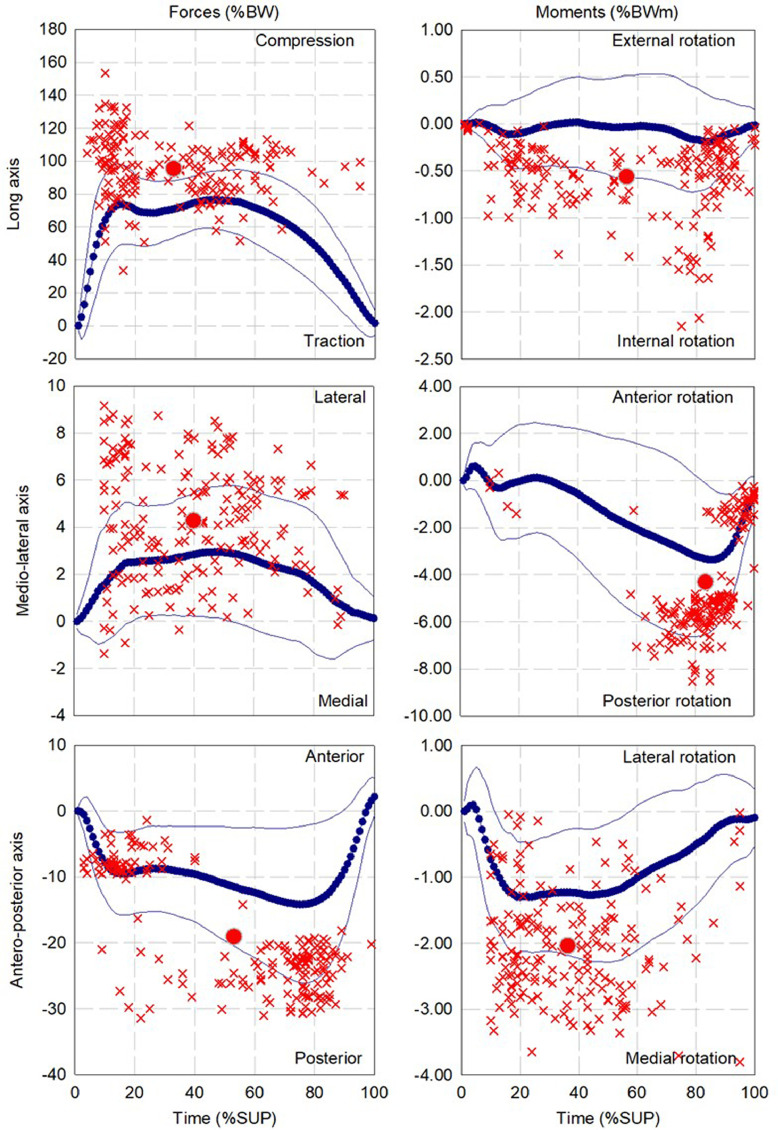
Dispersion (cross) and average (circle) for first (red), second (Blue) and third (green) local extrema of forces and moments for cohort of participants fitted with components (12 participants, 180 gait cycles) during descending stairs.

#### Characteristics of local extrema

**Fig. 11:**
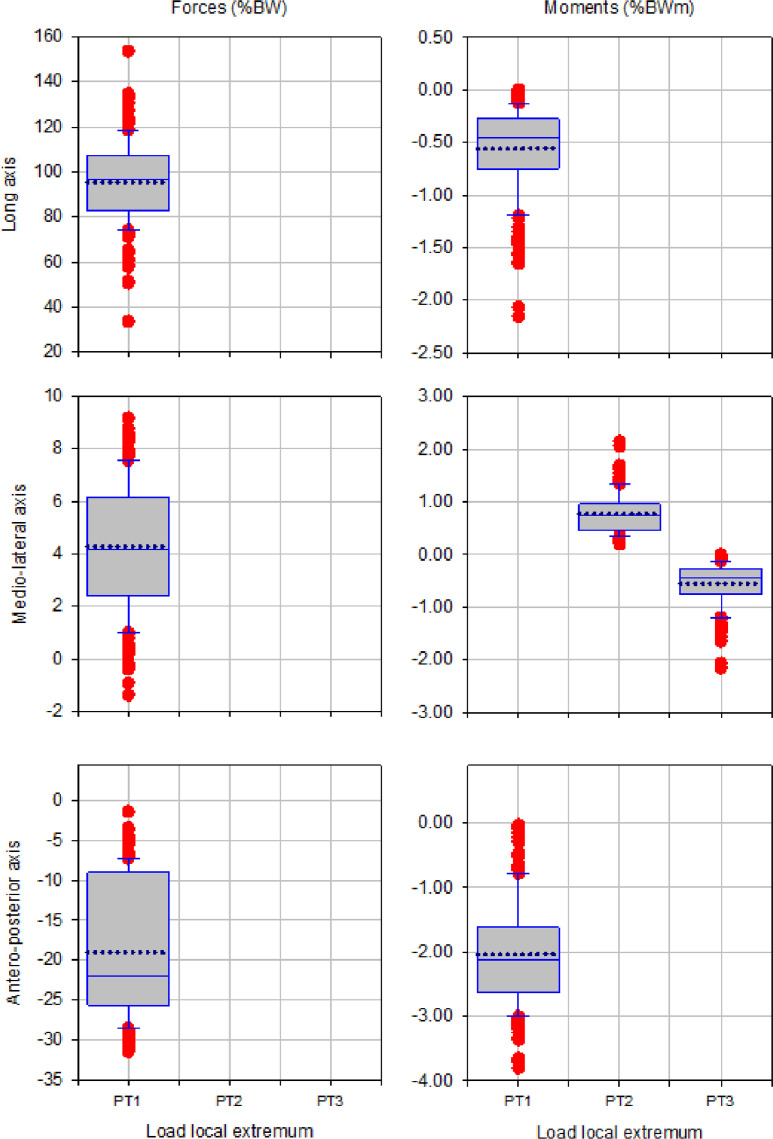
Box plots showing low and high 95% confidence interval, mean and outliers of the magnitude of up to three local extrema (PT1, PT2, PT3) of forces and moments applied with state-of-the-art components during descending stairs.

## 2 Experimental Design, Materials and Methods

### 2.1 Design

The study was designed as cross-sectional cohort study.

### 2.2 Participants

Thirteen participants with a single above-knee amputation fitted with press-fit osseointegrated implant participated in this study (Tables 1–3). They ambulated with a bone-anchored prosthesis. We estimated that this group corresponded to approximately 1.3% of the population of individuals with transfemoral amputation fitted with bone-anchored prostheses at the time of the recording, worldwide.

### 2.3 Prostheses

Participants ambulated with a bone-anchored prosthesis equipped with their own footwear, Pro-Flex XC or LP feet (ÖSSUR, Iceland), Rheo Knee XC (ÖSSUR, Iceland), iPecsLab's transducer (RTC Electronics, USA), and of tube and/or offset connector (Table 4) [18].

The Rheo Knee XC is a microprocessor-controlled knee. The Pro-Flex XC or LP feet are energy-storing-and-returning feet. These components are referred to as “state-of-the-art”. All Rheo Knee XC, Pro-Flex XC or LP feet are amongst the components frequently prescribed to individuals with osseointegrated implant worldwide, particularly in Australia [19].

The tri-axial transducer of the iPecsLab was inserted between the participant's offset adapter and knee unit. It measured load data at sampling frequency set at 200 Hz and sent the data wirelessly to a laptop close by (Tables 4 and 5). Forces and moments applied on mediolateral, anteroposterior and long axes of the implant were measured directly with an accuracy better than 1 N and 1 Nm, respectively [15,20,21].

All the percutaneous and medullar parts of the implant and the tube and/or connector were considered as a single rigid part. Nonetheless, the co-linearity of both long axes of the implant and the transducer varied according to the offset of the adapter (Tables 6 and 7, Fig. 1,).

### 2.4 Recording

Participants conducted a maximum of five trials of standardised daily activities, namely straight-line level walking, ascending and descending ramp and stairs (Table 8) [5,22]. Participants were asked to complete each activity at a self-selected speed. They could use the handrails. Sufficient rest between trials was allowed to avoid fatigue when required.

### 2.5 Loading characteristics

The raw load data (e.g., forces and moments) recorded by the transducer were imported and processed into a specifically designed Matlab program (The MathWorks, Inc, USA) [4,16].

The load data was extracted through the following steps:

1. Calibration. The raw data were offset depending on the magnitude of the load recorded during calibration recording.
2. Detection of relevant segment. The first and the last strides recorded were eliminated to avoid the effects of gait initiation and termination so that the analysis included only steps taken at a steady pace.
3. Detection of gait events. Each heel contact and toe-off event was detected manually using loading profile applied on the long axis.
4. Time normalization. Loading data were time-normalization from 0 to 100 throughout the support phase.
5. Bodyweight normalization. Loading data were expressed as percentage of bodyweight [4].

More advanced processing was required to characterize loading profile for each activity. This included extraction of loading patterns, loading boundaries (e.g., minimum and maximum of loading data across all gait cycles independently of the onset) and no more than three loading local extrema (e.g., onsets (%SUP) and magnitudes (%BW or %BWm) of points of inflection between loading slopes occurring consistently over successive gait cycles across all trials detected semi-automatically [1,4].

A loading pattern was described by its mean and one standard deviation. We reported confidence intervals calculated using the CONFIDENCE function in Microsoft Excel 2010 and the box plot showing low and high 95% confidence interval, mean and outliers created using SigmaPlot 11 (Systat Software, Inc, USA) for all discrete datasets (e.g., loading boundaries, local extrema) [14].

## Data availability

Loading data applied on osseointegrated implant by transfemoral bone-anchored prostheses fitted with state-of-the-art components: confounders and loading boundaries (Original data) (Mendeley Data).

## Ethics Statement

Each participant signed a written ethical consent form approved by research organization's human ethics committee (Human Research Ethics Committee Certificate No 1600000332, Queensland University of Technology, Brisbane, Australia).

## Transparency Document. Supporting Information

The data provided in Table 1, Table 2, Table 3, Table 4, 6, Table 8, Table 9, Table 10 in this article can be found in the online version at https://data.mendeley.com/datasets/gmsyv97cpc/1

## CRediT authorship contribution statement

**Laurent Frossard:** Conceptualization, Methodology, Software, Formal analysis, Investigation, Data curation, Writing – original draft, Writing – review & editing, Visualization, Project administration. **Stefan Laux:** Conceptualization, Methodology, Investigation, Resources, Supervision, Project administration, Funding acquisition. **Marta Geada:** Conceptualization, Methodology, Investigation, Resources, Funding acquisition. **Peter Paul Heym:** Conceptualization, Software, Validation, Formal analysis, Data curation, Writing – original draft, Writing – review & editing, Visualization. **Knut Lechler:** Conceptualization, Methodology, Resources, Writing – original draft, Writing – review & editing, Supervision, Project administration, Funding acquisition.

## Declaration of Competing Interest

The authors declare that they have no known competing financial interests or personal relationships that could have appeared to influence the work reported in this paper.
